# Transition of Temporal Scaling Behavior in Percolation Assisted Shear-branching Structure during Plastic Deformation

**DOI:** 10.1038/srep45083

**Published:** 2017-03-22

**Authors:** Jingli Ren, Cun Chen, Gang Wang, Peter K. Liaw

**Affiliations:** 1 School of Mathematics and Statistics, Zhengzhou University, 100 Science Road, Zhengzhou, 450001, China; 2 Laboratory for Microstructures, Institute of Materials, Shanghai University, Shanghai, 200444, China; 3 Department of Materials Science and Engineering, The University of Tennessee, Knoxville, TN, 37996, USA

## Abstract

This paper explores the temporal scaling behavior induced shear-branching structure in response to variant temperatures and strain rates during plastic deformation of Zr-based bulk metallic glass (BMG). The data analysis based on the compression tests suggests that there are two states of shear-branching structures: the fractal structure with a long-range order at an intermediate temperature of 223 K and a larger strain rate of 2.5 × 10^−2^ s^−1^; the disordered structure dominated at other temperature and strain rate. It can be deduced from the percolation theory that the compressive ductility, *e*_c_, can reach the maximum value at the intermediate temperature. Furthermore, a dynamical model involving temperature is given for depicting the shear-sliding process, reflecting the plastic deformation has fractal structure at the temperature of 223 K and strain rate of 2.5 × 10^−2^ s^−1^.

The plastic deformation behavior of bulk metallic glasses (BMGs) is receiving a considerable amount of attention^1^,^2^,^3^,^4^,^5^,^6^. It is well known that the mechanical properties of BMGs strongly depend on microstructures. The cryogenic temperature is one of important factors influencing the microstructure of BMGs^7^, which can further affect mechanical properties, such as the improvement of yield strength and plasticity^8^,^9^. Klaumünzer *et al*. studied temperature-dependent shear band dynamics during inhomogeneous deformation of Zr-based BMGs at temperatures between 233 K and 333 K^10^. It was reported that macroscopic change in the flow behavior was directly related to the strain rate and temperature in BMGs^11^,^12^. Chen *et al*. demonstrated a crossover between fractal short-range and homogeneous long-range structures^13^. In ref. 14, Zeng *et al*. reported a long-range topological order in the Ce_75_Al_25_ metallic glass. For the Zr-based BMG, a dynamical transition from chaotic to a critical state occurred with decreasing temperature from room temperature to cryogenic temperature^15^,^16^,^17^. In ref. 18, it was found that the plastic dynamics manifests a self-similar random process as the temperature changing. Noting that the strain rates also influence the plastic dynamics, we study the temporal scaling behavior during the shear-branching process at different strain rates and temperatures. By investigating the different temporal scales of order in the shear-branching process, we provide a new point of view on the fractal mechanism of the plastic deformation corresponding to the temperature and strain rate.

The analysis based on the experimental results manifests that the decrease of the temperature together with the increase of the strain rates induces a fractal behavior during the shear-branching process. We find that there exists self-similar scaling behavior only on narrow scale of zone, suggesting the fractal in the short range. At an intermediate temperature of 223 K with a larger strain rate of 2.5 × 10^−2^s^−1^, the signal shows self-similar scaling behavior on broader scale of zones, suggesting that there is a fractal behavior with a longer range of correlation. Meanwhile, the percolation theory is introduced to interpret the mechanism of fractal structure at a critical temperature. The dynamic model involving temperature is established, and the statistics based on the numerical simulation show that the stress drops obey power law distribution at an intermediate temperature with a larger strain rate, which also reflects the fractal structure of the shear bands.

A Zr_64.13_Cu_15.75_Al_10_Ni_10.12_ (at. %) BMG is compressed in the temperature range from 193 K to 293 K with a strain rate of 2.5 × 10^−4^ s^−1^, and different strain rates of 2.5 × 10^−4^ s^−1^, 2.5 × 10^−3^ s^−1^, and 2.5 × 10^−2^ s^−1^ at the temperatures of 293 K, 223 K, and 203 K, respectively. The morphologies of shear bands on the lateral surface of the fractured sample are shown in Fig. 1. The compressive nominal stress-time curves are shown in Fig. 2(a). It is clearly seen that at the temperature of 293 K and 273 K there is a transition from branched shear banding regime to a singular stick-slip type shear in a dominant shear band, which is consistent with the result in ref. 19. Moreover, from Fig. 2(a), the serration is weaker at lower temperature, which is consistent with the earlier work that the serrations disappear at cryogenic temperature^20^,^21^. The global temporal-scaling behavior is explored by applying the detrended fluctuation analysis (DFA)^22^,^23^,^24^,^25^, which reflects the shear-branching structure during the plastic deformation. Therefore, the following analysis will focus on the plastic deformation regime after yielding. Figure 2(b) sketches the determination of the yield point. After the stress is increased to be higher than the yield stress, the plastic deformation commences. The deformation regime from the yield point to the fracture is focused.

The detrended fluctuation analysis is based on the stress-rate signal, 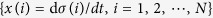. Divide the data of 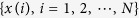 into *N*_*q*_ zones with each zone containing *q* elements (where *N*_*q*_ = *N/q*). For the *k*-th zone, the local trend is defined as a linear function of 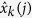, 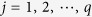, which is fitted from *x*_*k*_(*j*), *j* = 1, 2, ···, *q*. Define 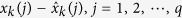 as the detrended time series, with the mean-square error, 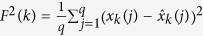, and the root-mean-square 
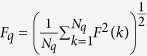
. *F*_*q*_ is a power function of the scale, *q, F*_*q*_~*q*^*H*^, here *H* is the Hurst exponent, reflecting the long-range correlation of time series.

Differing from the conventional detrended fluctuation analysis, we focus on the scaling behavior in different scales of *q*, where *q* is defined as the temporal scale. The scaling relation for different temperatures and strain rates are plotted in Figs 3 and 4, respectively. For fixed strain rate 2.5 × 10^−4^ s^−1^, such as at the temperature of 293 K, there exists a linear relation on small temporal scales of *q*, which corresponds to the short-range correlation [see Fig. 3(a)]. When the scale, *q*, increases, the self-similar behavior disappears, and then the scale-free behavior only exists in several narrow ranges of *q*. When the temperature decreases to 223 K, the scale-free behavior mainly exists in two zones of the temporal scale [see Fig. 3(c)]. With decreasing the temperature, scale-free behavior becomes complicated again [see Fig. 3(d,e) and (f)].

At a fixed temperature, such as at the temperature of 223 K, when the strain rate increases from 2.5 × 10^−4^ s^−1^ to 2.5 × 10^−2^ s^−1^, the scaling relation becomes more and more obvious for wide range of temporal scales (see Fig. 4). At the larger strain rate of 2.5 × 10^−2^ s^−1^, there is a most obvious scale-free relation in the wide range of temporal zone [see Fig. 4(f)], which is the characteristic of fractal.

The scaling behavior varies as temporal scales changing from short-range to long-range, which inevitably induces the transition in the shear-branching structure. From Fig. 4, at the temperatures and the strain rate except the turning temperature 223 K with the strain rate of 2.5 × 10^−4^ s^−1^, there is only scale-free behavior in some narrow scales of zones, i.e. the fractal behavior is broken in the long temporal range [such as Fig. 4(a)], reflecting that there is no global fractal behavior in the long range, and meaning that the shear branching process is complicated in the temporal medium-range or long-range. The broken scaling behavior on the global temporal scales of *q* means the disordered structure in the long range. Meanwhile, when the temperature decreases and/or the strain rate increases, the global scaling behavior is the most obvious at the intermediate temperature of 223 K and a larger strain rate of 2.5 × 10^−2^s^−1^ [see Fig. 4(f)], at which the structure evolves in a manner of a self-similar fractal. Therefore, it is evident that the fractal structure with a scale-free behavior bursts at a critical temperature with a large strain rate.

Although the serrated flow dynamics in the BMGs is also influenced by the strain rate^6^, note that the strain rate ranges in a narrow zone from ~10^−4^ s^−1^ to ~10^−2^ s^−1^, there is no sufficient data to show the transition of the scaling behavior at an intermediate strain rate. Figure 4 shows that the turning temperature cannot be significantly influenced by the strain rate. It also will remain further discussing about the transition of the scaling behavior at the intermediate temperature. Considering the fractal structure of the shear branching is percolation assisted, as such, the percolation theory is used to further explore the scaling transition at the turning temperature.

Based on the composite mechanics^26^, the yield strength of the glass is dependent on *V*_*α*_:


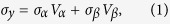


where *V* and *σ* represent the volume fraction and yield strength of the constituents, and the subscripts, *α* and *β* refer to the crystalline phase and BMG phase, respectively. The microstructure transition in the shear-branching process is considered to be related to a critical volume fraction, at which the yield strength drops suddenly^27^. With increasing the crystalline volume fraction, the interpenetration continues and forms a structural framework, which is considered as percolation process quantifying the formation of the long-range connectivity in random systems^28^. The compressive ductility, *e*_*c*_, can be quantitatively modeled by applying the percolation theory. 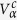 represents the critical volume fraction referring to the crystalline phase. For 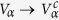, the compressive ductility diverges as a power-law distribution with an exponent of -*ν* in terms of the distance of *V*_*α*_ from 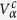, i.e., 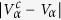, that is,


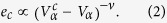


For comparison, the yield strength changes linearly as a function of temperature^29^, that is, *σ*_*y*_ = *aT + b*, where *T* is the temperature, and the coefficients, *a* and *b*, can be linearly fitted based on the experimental data. The relationship between *V*_*α*_ and *σ*_*y*_ involves the influence of the temperature, *T*,


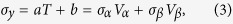


It can be deduced that, 
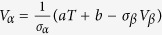
, and then,


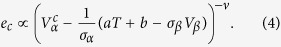


Set *V*_*β*_ as a determined parameter, here we regard *T* as a variable. Thus, 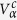 can be presented as 
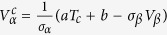
, and *T*_*c*_ is denoted as the intermediately critical temperature at which a transition microstructure of the shear-branching process appears. Considering the unimodal hump-shaped curve of *e*_*c*_, at a critical temperature, *T*_*c*_, the compressive ductility, *e*_*c*_, reaches the maximum value.

The above qualitative analysis reflects the characteristic of the self-similar fractal behavior in the plastic deformation, based on which we draw a question: how to construct a bridge to link shear-banding process and dynamical behavior in the plastic dynamics of BMGs. We present a sliding shear-displacement (SSD) model to describe the shear-banding dynamics at different temperatures and different strain rates. A special case of the SSD model at room temperature has been discussed in ref. 30. The model in the present work involving the temperature contains a chain of shear blocks coupled to each other by harmonic springs with strength of *k*_*c*_. The chain of shear blocks is attached to the testing machine with strength of *k*. The system is compressed at a loading speed, *v*. The motion equation is


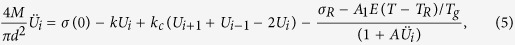


where *U*_*i*_ is the shear-sliding displacement of the *i-*th block, *σ*(0) is the initial internal stress that is equivalent to the yield stress at room temperature, 
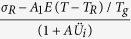
 represents the shear resistance along the shear plane^31^,^32^ (where *T* is the environmental temperature, *T*_*R*_ is the room temperature, *σ*_*R*_ is the yield stress at room temperature, *T*_*g*_ is the glass-transition temperature.), *d* is the diameter of the sample, *M* is the equivalent mass of the system (which consists of a BMG and a spring that represents the influence of the testing machine), *k* = *E/L*(1 + *S*) (here, *E* is the Young’s modulus of the metallic glass, *L* is the length of the sample, and *S* is the stiffness ratio of the sample, *κ*_s_, to the testing machine, *κ*_M_, i.e., *S* = *κ*_s_/*κ*_M_ = *πd*^2^/*E*/4 *Lκ*_M_)^33^.

The numerical simulation based on equation (5) is resolved with the periodic boundary conditions of *U(x*, 0) = 0, *U*(0, *t*) = *U(L, t*), *U*_*t*_(*x*, 0) = *v* (Set *ν* = *ε· L*, where *ε* is the strain rate), which are shown in Fig. 5(a,b and c) at different temperatures of 293 K, 223 K, and 203 K with a strain rate of 2.5 × 10^−4^s^−1^ and Fig. 5(d) at a temperature of 223 K with a strain rate of 2.5 × 10^−2^ s^−1^. The figures are plotted in the form of the sliding velocity of the shear blocks, 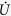, as a function of position, *i*, and time, *t*. Based on the numerical results, the statistics of the sliding speed of the *i-*th blocks, 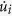 (here 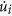 is denoted by *s* for convenience) at the temperature of 223 K show a power-law distribution with a fitting exponent of *α* = 2.1 [see Fig. 6]. Noting that Δ*σ* = *k(s−ν*)Δ*t*. The stress drops also behave as a power-law distribution at the temperature of 223 K and the strain rate of 2.5 × 10^−2^ s^−1^, which reflecting a scale-free behavior in the plastic fracture. In addition, the continuous decrease of the temperature causes the self-similar behavior disappearing, and the shear-branching structure will undergo a transition at a turning temperature of 223 K.

To sum up, in different temporal zones, there is a transition of the temporal scaling behavior at an intermediate temperature of 223 K and a larger strain rate of 2.5 × 10^−2^ s^−1^, which inevitably induces the transition in the shear-branching process during the plastic deformation with increasing the temperature. Meanwhile, the fractal structure at the critical temperature is the percolation associated. The self-similarity in global temporal zones suggests an ordered structure of the shear-branching process. The short-range correlation means a disordered shear-branching process in the global zone, which corresponds to the homogeneous structure. In addition, a phenomenological dynamical model involving temperature is developed, suggesting that the process of the shear blocks slide shows a critical state at the intermediate temperature, also reflecting the fractal structure of the shear-branching process. In this paper we have given explicit theoretical support on how the fractal burst at a certain condition, which can address the intrinsic mechanism of the shear-banding process corresponding to the intermediate state.

## Additional Information

**How to cite this article:** Ren, J. *et al*. Transition of Temporal Scaling Behavior in Percolation Assisted Shear-branching Structure during Plastic Deformation. *Sci. Rep.* **7**, 45083; doi: 10.1038/srep45083 (2017).

**Publisher's note:** Springer Nature remains neutral with regard to jurisdictional claims in published maps and institutional affiliations.

## Figures and Tables

**Figure 1:**
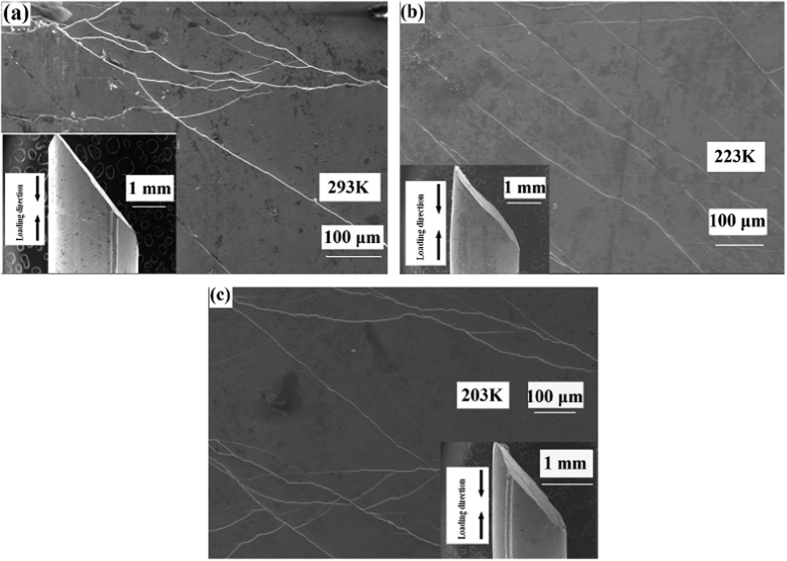
Surface morphologies of shear bands on the lateral surfaces for the fractured Zr_64.13_Cu_15.75_Al_10_Ni_10.12_ glassy metals compressed with a strain rate of 2.5 × 10^−4^ s^−1^ at different temperatures. (**a**) 293 K. (**b**) 223 K. (**c**) 203 K.

**Figure 2:**
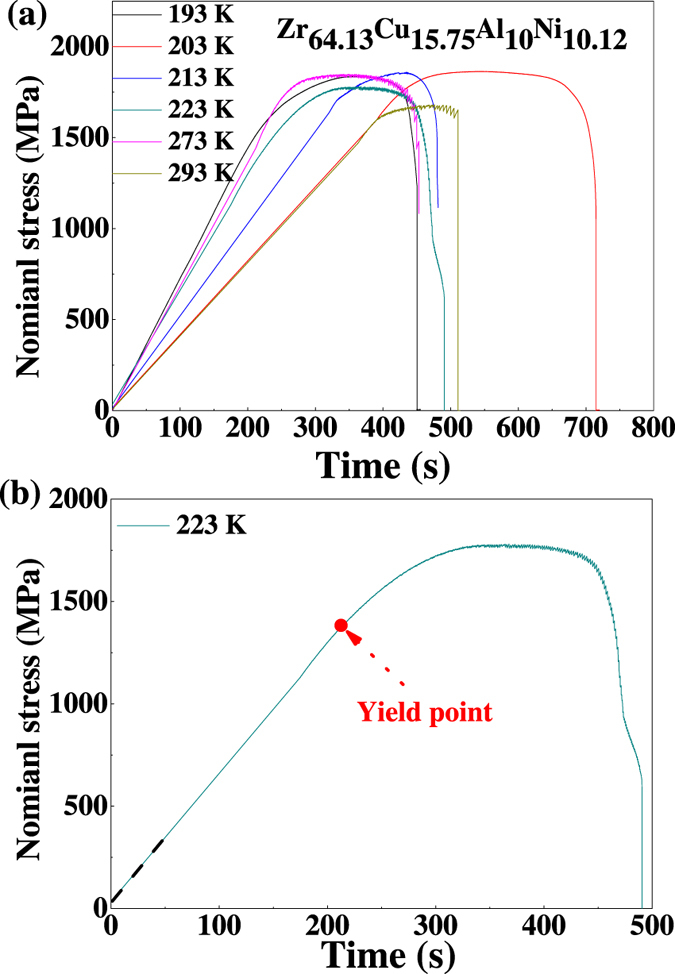
(**a**) Comparative stress-time curves for the Zr_64.13_Cu_15.75_Al_10_Ni_10.12_ glassy metal deformed at different temperatures, 193 K, 203 K, 213 K, 223 K, 273 K, and 293 K with a strain rate of 2.5 × 10^−4^ s^−1^. (**b**) Sketch of the yield point.

**Figure 3:**
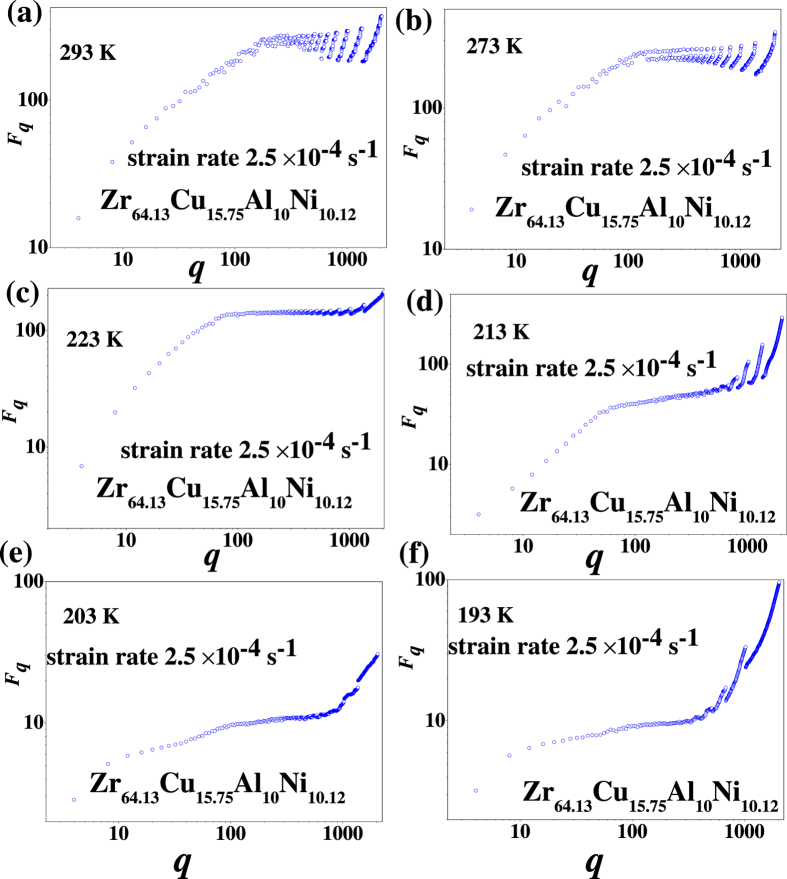
Temporal scale *q* vs *F*_*q*_ in double-logarithmic coordinate at different temperatures of (**a**) 293 K, (**b**) 273 K, (**c**) 223 K, (**d**) 213 K, (**e**) 203 K, and (**f**) 193 K with a strain rate of 2.5 × 10^−4^ s^−1^.

**Figure 4:**
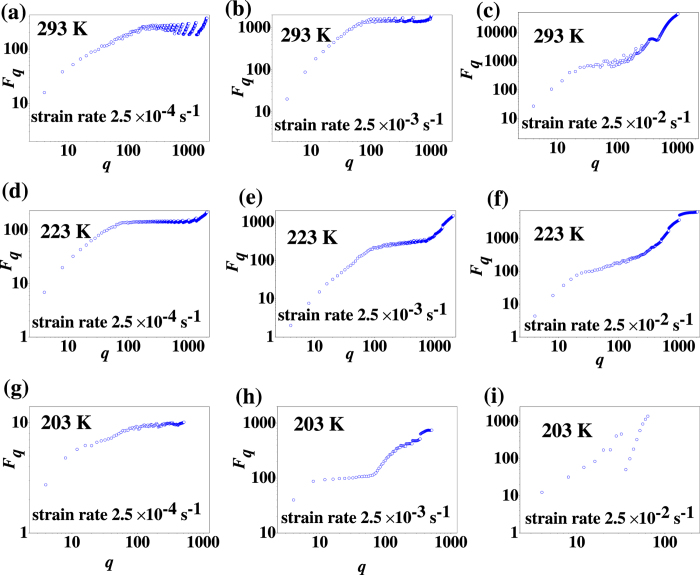
Temporal scale *q* vs *F*_*q*_ in double-logarithmic coordinate with different strain rates of (**a**) 2.5 × 10^−4^ s^−1^, (**b**) 2.5 × 10^−3^ s^−1^, (**c**) 2.5 × 10^−2^ s^−1^ at the temperature of 293 K; (**d**) 2.5 × 10^−4^ s^−1^, (**e**) 2.5 × 10^−3^ s^−1^, (**f**) 2.5 × 10^−2^ s^−1^at the temperature of 223 K; (**g**) 2.5 × 10^−4^ s^−1^, (**h**) 2.5 × 10^−3^ s^−1^, (**i**) 2.5 × 10^−2^ s^−1^at the temperature of 203 K.

**Figure 5:**
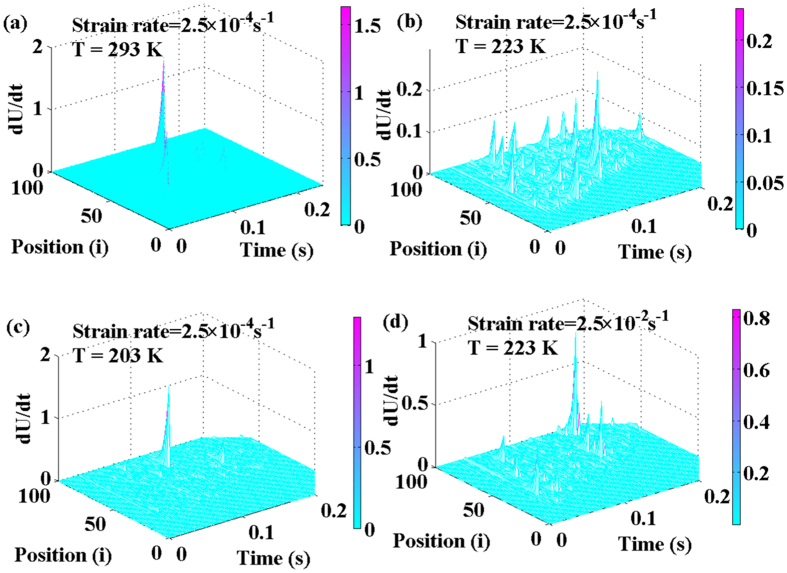
Numerical simulation at different temperatures with a strain rate of 2.5 × 10^−4^s^−1^, *N* = 100, (**a**) At a temperature of 293 K, (**b**) At a temperature of 223 K, (**c**) At a temperature of 203 K; (**d**) At a temperature of 223 K with a strain rate of 2.5 × 10^−2^ s^−1^. The parameters are *d* = 2 mm, *L* = 4 mm, *T*_*R*_ = 273 K, *T*_*g*_ = 643 K, *σ*_*R*_ = 1, 742 MPa, *A*_1_ = 0.0106, *E* = 66.7 GPa, and *M* = 20 kg.

**Figure 6:**
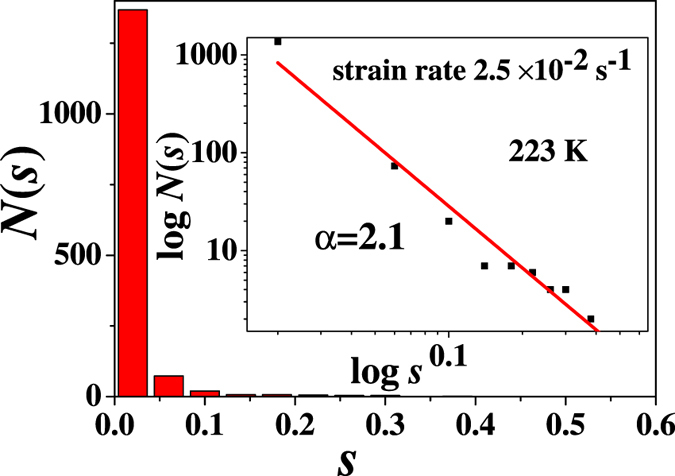
The distribution, *D(s***), vs. the sliding speed of each block** 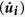 at the temperature of 223 K with a strain rate of 2.5 × 10^−2^ *s*^−1^, where *D(s*) represents the number of blocks with a sliding speed, *s*.
